# Hyperacute Interleukin‐1β Production and Neutrophil Extracellular Trap Formation in the Cerebral Circulation of Stroke Patients with Large Vessel Occlusion

**DOI:** 10.1002/ana.78284

**Published:** 2026-06-22

**Authors:** Justine Münsterberg, Caspar Brekenfeld, Hanna Englert, Marius Piepke, Regine J. Dress, Martin Oertel, Tina Fischer, Romy Hackbusch, Mingming Zha, Haodi Cai, Christian Casar, Ines S. Schädlich, Leo Winter, Alina Jander, Karoline Degenhardt, Maxim Bester, Fabian Flottmann, Uta Hanning, Brigitte Holst, Anna Worthman, Johanna Hiefner, Berenike Rutz, Eckhard Schlemm, Götz Thomalla, Bettina H. Clausen, Ann M. Stowe, Immo Prinz, Thiruma V. Arumugam, Markus Glatzel, Thomas Renné, Jens Fiehler, Eva Tolosa, Tim Magnus, Mathias Gelderblom

**Affiliations:** ^1^ Department of Neurology University Medical Center Hamburg‐Eppendorf Hamburg Germany; ^2^ Department of Neuroradiology University Medical Center Hamburg‐Eppendorf Hamburg Germany; ^3^ Institute of Clinical Chemistry and Laboratory Medicine University Medical Center Hamburg‐Eppendorf Hamburg Germany; ^4^ Institute of Systems Immunology University Medical Center Hamburg‐Eppendorf Hamburg Germany; ^5^ Department of Immunology University Medical Center Hamburg‐Eppendorf Hamburg Germany; ^6^ Bioinformatics Core University Medical Center Hamburg‐Eppendorf Hamburg Germany; ^7^ Department of Biochemistry and Molecular Cell Biology University Medical Center Hamburg‐Eppendorf Hamburg Germany; ^8^ Department of Neurobiology Research, Institute of Molecular Medicine University of Southern Denmark Odense Denmark; ^9^ Department of Neuroscience University of Kentucky Lexington KY USA; ^10^ Hamburg Center for Translational Immunology University Medical Center Hamburg‐Eppendorf Hamburg Germany; ^11^ Centre for Cardiovascular Biology and Disease Research La Trobe University Melbourne VIC Australia; ^12^ Institute of Neuropathology University Medical Center Hamburg‐Eppendorf Hamburg Germany

## Abstract

**Objective:**

Cerebral ischemia remains a major cause of disability, and the contribution of the hyperacute immune response is increasingly recognized. The aim of this study was to investigate the local inflammatory response in the affected brain of stroke patients with large vessel occlusion during the hyperacute phase of stroke.

**Methods:**

To decipher the role of myeloid immune cells in stroke‐induced inflammation, we performed an unsupervised multiomics analysis of innate immune pathways in ischemic blood obtained directly from the occluded middle cerebral artery in 54 patients undergoing mechanical recanalization. Paired nonischemic arterial blood served as an internal control.

**Results:**

Stroke triggered a local hyperacute activation of classical monocytes and neutrophils in the ischemic cerebral vasculature, with interleukin (IL)‐1β emerging as a key mediator of inflammation. Elevated plasma adenosine triphosphate levels and inflammasome priming in intravascular monocytes were associated with IL‐1β production within the occluded middle cerebral artery within 4.5 hours of stroke onset. IL‐1β release coincided with increased neutrophil‐attracting chemokines (C‐X‐C motif chemokine ligand 1 [CXCL1], IL‐8). Locally activated neutrophils formed neutrophil extracellular traps (NETs) within the ischemic vasculature, a hallmark of thromboinflammation. Postmortem analyses revealed NET deposition within ischemic brain parenchyma.

**Interpretation:**

These findings indicate increased IL‐1β expression and enhanced NET formation within the cerebral circulation in stroke caused by large vessel occlusion, suggesting that these mechanisms might contribute to early stroke pathophysiology and represent potential targets for immunomodulatory strategies. ANN NEUROL 2026;100:824–840

## Graphical abstract

Acute ischemic stroke due to large vessel occlusion is associated with rapid intravascular immune activation. Analysis of arterial blood sampled distal to the thrombus during mechanical thrombectomy revealed increased extracellular adenosine triphosphate (ATP) and interleukin‐1β (IL‐1β) levels, evidence of inflammasome priming in monocytes, and elevated concentrations of neutrophil‐attracting chemokines (C‐X‐C motif chemokine ligand 1 [CXCL1], interleukin‐8 [IL‐8]). These changes were accompanied by increased markers of neutrophil extracellular trap formation. Together, these findings describe a hyperacute intravascular inflammatory signature in human ischemic stroke.

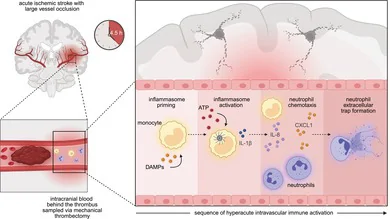

Ischemic stroke is one of the leading causes of death and disability worldwide.^1^ Since 2015, mechanical thrombectomy has complemented intravenous thrombolysis for reperfusion therapy of large vessel occlusions (LVO).^2^ However, despite successful recanalization, >30% of patients with LVO develop large infarct cores, leading to severe long‐term disability.^3^ Because excessive inflammation is recognized as a key element for the secondary infarct growth in the acute stroke phase, additive immunomodulatory therapies represent a promising option for patients with large vessel occlusions.

Deleterious postischemic inflammation in the brain is triggered by danger signals released from dying cells,^4^ and orchestrated by both brain‐resident microglia and peripheral immune cells, which are rapidly recruited to the injury site.^5^ The proinflammatory cytokine, interleukin‐1β (IL‐1β), produced by monocytes and microglia,^6^ is pivotal for the postischemic immune response. In animal models, IL‐1β exacerbates inflammation in the ischemic brain by recruiting and activating neutrophils.^7^ Activated neutrophils exert damaging effects in both the intra‐ and perivascular space of the cerebral vascular system in experimental models of stroke by forming neutrophil extracellular traps (NETs).^8^, ^9^, ^10^ By interacting with platelets and the plasmatic coagulation cascade, NETs contribute to the ischemic injury through thrombus formation and microcirculatory impairment,^11^, ^12^ a process known as thromboinflammation. Experimental studies have demonstrated that targeting neutrophils and IL‐1β with immunotherapies significantly reduces the interplay between inflammatory and thrombotic pathways, and improves neurological outcomes.^13^, ^14^, ^15^

Therefore, we hypothesize that, particularly in strokes with large ischemic infarct cores, cell death leads to a hyperacute excessive activation of innate immune pathways in the brain vasculature. Yet, the translation of mechanistic findings from experimental studies to human stroke has proven difficult, as the analysis of systemic blood does not reflect hyperacute local immunity in the ischemic brain. To address this limitation, we performed a state‐of‐the‐art multiomic characterization of ischemic blood samples obtained during mechanical thrombectomies from the occluded segment of the middle cerebral artery in acute stroke patients with LVO.

The paired analysis of ischemic samples and nonischemic arterial samples from the ipsilateral carotid artery provides an unprecedented insight into hyperacute immunity in the human brain. Using this approach, we found that immune cells from the intravascular space contribute to the postischemic immune response within the first few hours after stroke onset. Local production of IL‐1β and NET formation represent key hallmarks of the hyperacute intravascular immune reaction. Our analysis of liquid biopsies from the ischemic brain of acute stroke patients underscores the potential of targeted inhibition of the IL‐1β‐driven immune cascade to prevent harmful thromboinflammation and reduce the neurotoxic propagation of intravascular inflammation into brain tissue. Hence, we provide a valuable resource for clinical studies on immunotherapies as an additive treatment to established recanalization procedures for patients with large infarct cores caused by LVO.

## Methods

### Study Design

**FIGURE 1:**
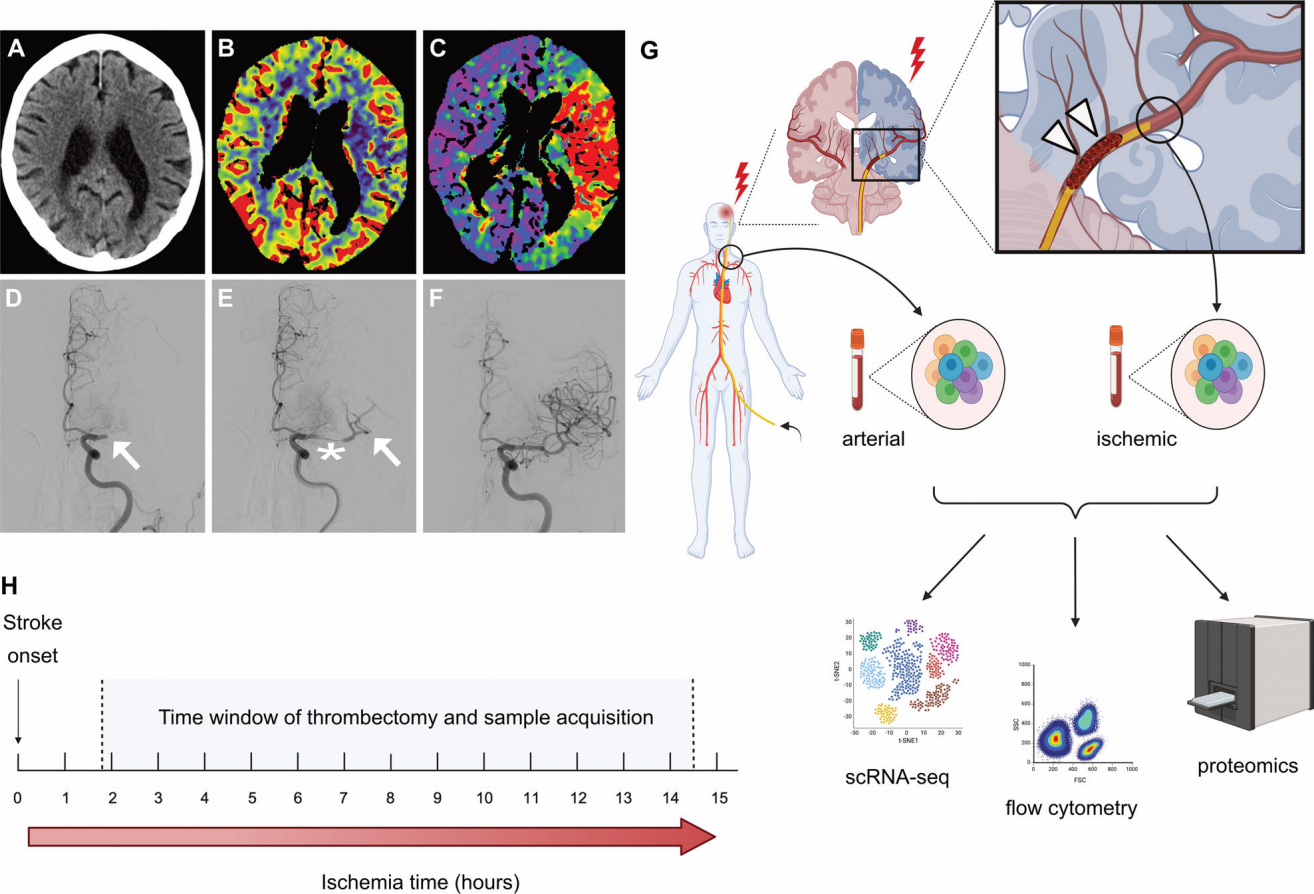
Sample acquisition workflow. Cerebral computed tomography imaging of an exemplary patient with acute stroke secondary to large vessel occlusion. Although the (A) native computed tomography is unremarkable, computed tomography perfusion imaging already shows the (B) infarct core (reduction in cerebral blood volume) and the (C) ischemic penumbra (lengthening of the time to peak) in the territory of the left middle cerebral artery (MCA). (D) Cerebral angiogram confirms MCA occlusion (arrow). (E) Position of the microcatheter, that was advanced through the thrombus into the occluded part of the MCA (*). The ischemic blood sample is aspirated with the microcatheter out of the occluded MCA (arrow). (F) Complete MCA recanalization after thrombus removal. (G) Schematic representation of catheter position (yellow line) distal to the thrombus (triangles) and sample collection. The first sample (ischemic) is aspirated from the occluded MCA distal to the thrombus before retrieval of the thrombus. During catheter retraction, a second sample is acquired from the ipsilateral carotid artery as a nonischemic internal control (arterial). (H) Timeline of sample acquisition (in hours) relative to stroke onset. scRNA‐seq = single‐cell RNA sequencing. [Color figure can be viewed at www.annalsofneurology.org]

The goal of this study was to investigate the hyperacute detrimental postischemic immune response in patients with LVO using a translational liquid biopsy approach. To this end, we prospectively enrolled 54 patients with acute ischemic stroke caused by LVO of the middle cerebral artery within 24 hours after stroke onset. Patient characteristics are listed in Table 1. All patients underwent mechanical thrombectomy at the University Medical Center Hamburg‐Eppendorf (Hamburg, Germany) between 2020 and 2024. Acute vessel occlusion was confirmed before endovascular treatment by multimodal computed tomography or magnetic resonance imaging. Details on inclusion criteria are provided in the Supplementary Methods. Blood samples were collected distal (ischemic blood) and proximal (nonischemic arterial blood) to the thrombus (Fig 1). If applicable, a separate peripheral venous sample was taken. A total of 10 nonstroke arterial control blood samples were collected from the internal carotid artery from patients undergoing diagnostic angiography (Supplementary Table S1). Ethical approval for this study was granted by the Ethical Review Board of Hamburg (PV7326). All patients provided written informed consent. Additional brain autopsy tissue from 3 patients was obtained and utilized in accordance with ethical approval (2022‐100885‐BO‐ff).

**Table 1:** Demographics and Characteristics for Thrombectomy Subjects

Patients’ characteristics (total n = 54)^a^	Group A: Cytokine analysis (n = 29)	Group B: NETosis analysis (n = 33)	Group C: ATP analysis (n = 10)
Age (years), mean (range)	74 (46–92)	71 (46–92)	70 (48–90)
Sex			
Female	13 (45%)	17 (52%)	5 (50%)
Male	16 (55%)	16 (48%)	5 (50%)
Comorbidities			
Hypertension	20 (69%)	23 (70%)	7 (70%)
Type 2 diabetes	7 (24%)	12 (36%)	1 (10%)
Dyslipidemia	17 (59%)	19 (58%)	5 (50%)
Previous cardiovascular events	9 (31%)	11 (33%)	1 (10%)
NIHSS^b^ score on admission			
Minor stroke (NIHSS 1–4)	1 (3%)	0 (0%)	2 (20%)
Moderate stroke (NIHSS 5–15)	15 (52%)	18 (55%)	4 (40%)
Severe stroke (NIHSS 16–20)	8 (28%)	8 (24%)	1 (10%)
Very severe stroke (NIHSS ≥21)	5 (17%)	7 (21%)	3 (30%)
i.v. thrombolysis treatment	17 (59%)	19 (58%)	6 (60%)
ASPECTS^c^ on admission, median (range)	8 (4–10)	7 (4–10)	8 (4–10)
Ischemia time^d^ (min), mean (range)	277 (97–609)	284 (97–787)	431 (125–876)

^a^ N = 18 patients from group A and B overlap.

^b^ The National Institutes of Health Stroke Scale (NIHSS) score assesses the severity of stroke syndrome, ranging from 0, indicating no deficits, to 42, representing the most severe condition.

^c^ The Alberta Stroke Program Early CT score (ASPECTS) is a 10‐point quantitative topographical score used to measure the extent of early ischemic changes on computed tomography scans. It ranges from 0, where all specified regions show early ischemic changes, to 10, where none of the specified regions show early ischemic changes.

^d^ Stroke onset to thrombus passage/recanalization time; not applied for wake‐up strokes with unknown time of symptom onset (group A: n = 8; group B: n = 12; group C: n = 2).

### Sample Acquisition Protocol

Detailed procedures for blood sampling are provided in the Supplementary Methods. Briefly, blood samples were obtained during mechanical thrombectomy (Fig 1). A guidewire was advanced via the internal carotid artery to the site of intracranial vessel occlusion, which was crossed with a microcatheter. A small‐volume arterial blood sample (**~**500 μL) was collected distal to the thrombus from the ischemic compartment before vessel recanalization. During catheter withdrawal, a systemic arterial blood sample was obtained from the proximal internal carotid artery or the aorta.

### Plasma Preparation

Whole blood was centrifuged at 480 *g* for 5 minutes, followed by a second centrifugation at 2,000 *g* for 10 minutes at room temperature (RT). Plasma was immediately frozen at −80°C until further analysis.

### Generation of Single‐Cell RNA Sequencing Libraries

Peripheral blood mononuclear cells were isolated from whole blood samples and stained with TotalSeq‐C Human Universal Cocktail (BioLegend; #399905). Single‐cell sequencing libraries were generated using Chromium Single Cell 5′ Reagent Kits v2 according to the manufacturer's protocol (10x Genomics). Indexed libraries were pooled, and paired‐end sequencing was performed (NovaSeq/Illumina).

### Computational Analysis

Downstream analyses were performed using Seurat (version 5.0.3; Satija Lab). Cell type annotation was conducted using the SingleR package (version 2.4.1; Bioconductor) with the Human Primary Cell Atlas^16^ reference dataset and manually validated based on marker gene expression. We filtered for monocytes, B‐, T‐, natural killer, and dendritic cells. Monocyte subgroups were annotated by the SingleR package with reference to the dataset MonacoImmuneData.^17^ Data visualization was performed using ggplot2 (version 3.5.1). Details are provided in the Supplementary Methods.

### Plasma Cytokine Analysis

The U‐Plex Custom Biomarker Assay (GROα [C‐X‐C motif chemokine ligand 1; CXCL1], IL‐1α, IL‐1β, IL‐1 receptor antagonist [IL‐1RA], IL‐4, IL‐8, IL‐10, tumor necrosis factor [TNF]α; #K15067M‐1; Meso Scale Discovery) was used according to the manufacturer's instructions. A detailed experimental description is provided in the Supplementary Methods.

### Ex Vivo Analysis of Intracellular Cytokine Production and Inflammasome Activation

For cytokine analysis, 100 μl whole blood was restimulated with lipopolysaccharide (1.25 μg/ml; #L6511; Sigma‐Aldrich) and interferon‐γ (100 ng/ml; #AF‐300‐02; PeproTech) for 8 hours at 37°C; for inflammasome induction, blood was treated with adenosine triphosphate (ATP; 2.5 mmol/l; #A6419; Sigma‐Aldrich) for 30 minutes at 37°C. Cells were surface stained (Supplementary Table S2), subjected to red blood cell lysis (#422401; BioLegend), permeabilized with 0.2% saponin (#84510; Sigma‐Aldrich), and stained intracellularly for cytokines (30 minutes, RT) or apoptosis‐associated speck‐like protein containing a CARD (ASC; 30 minutes, 4°C). Samples were acquired immediately by flow cytometry (FACSCelesta™, FACSDiva™; BD Biosciences), and ASC specks were identified by a reduced pulse width‐to‐area ratio. Details are provided in the Supplementary Methods.

### ASC Speck Imaging

Peripheral blood mononuclear cells were stimulated with ATP, as described above. 1 × 10^6^ cells/ml were spun down and stained with primary antibodies against CD14 (1:50; M5E2, #60004; Stemcell Technologies) and ASC (1:100; clone EPR23978‐28, #ab283684; Abcam) for 1.5 hours at RT, followed by fluorescent secondary antibody staining for 45 minutes at RT. Nuclei were stained with DAPI (#HP20.1; Carl Roth). The detailed experimental description can be found in the Supplementary Methods.

### Liquid Chromatography–Tandem Mass Spectrometry‐Based Targeted Metabolomics

ATP clearance inhibitor mix was added immediately after the blood was collected (Supplementary Table S3).^18^ Plasma metabolites were quantified by high‐performance liquid chromatography–tandem mass spectrometry, as previously described.^19^, ^20^ The detailed experimental description can be found in the Supplementary Methods.

### Neutrophil Flow Cytometry

A total of 50 μl of whole blood was incubated with a surface antibody cocktail (CD14 [M*φ*P‐9]; BD Biosciences; CD16 [3G8] and CD45 [2D1]; BioLegend; Supplementary Table S2) for 30 minutes at RT, followed by erythrocyte lysis (#422401; BioLegend) and immediate acquisition by flow cytometry (FACSCelesta™) using FACSDiva™ software (BD Biosciences).

### Citrullinated Histone H3 Enzyme‐Linked Immunosorbent Assay

Levels of citrullinated histone H3 (CitH3) in patient plasma were measured using a CitH3 enzyme‐linked immunosorbent assay kit (#501620; Cayman Chemical). Patient plasma diluted 1:10 was analyzed according to the manufacturer's protocol. Samples were run in duplicate on 96‐well microtiter plates and read at 450 nm using a microplate reader (Tecan Spark 10M).

### In Vitro NETosis Induction

For neutrophil isolation, peripheral venous blood was collected from healthy controls. Cells were prepared as previously described.^21^ The neutrophil isolation protocol can be found in the Supplementary Methods. A total of 2 × 10^5^ neutrophils/well were seeded in a 96‐well plate in Dulbecco's modified Eagle medium without phenol red (#31053028; Thermo Fisher) and incubated for 15 minutes at 37°C to adhere to the plate. Neutrophils were activated with 10% patient plasma or were left unstimulated for 4 hours at 37°C.^22^ To neutralize IL‐1β in the patient plasma, 1 ng/mL of interleukin‐1 receptor antagonist (#200‐01RA‐1MG; Thermo Fisher) or control substrate was added. After incubation, NET formation was assessed using a CitH3 enzyme‐linked immunosorbent assay.

### Immunofluorescence Analysis of NET Formation In Vitro

Stimulated neutrophils were seeded onto poly‐L‐lysin‐coated coverslips (#P4707; Sigma‐Aldrich) and permeabilized. Primary antibodies were applied for 1 hour at RT as previously described.^23^ The detailed experimental description can be found in the Supplementary Methods.

### Immunofluorescence NET Staining of Postmortem Brain Tissue

Paraffin sections were deparaffinized, rehydrated, and subjected to antigen retrieval in sodium citrate buffer (pH 6.0) for 20 minutes. Sections were then blocked with 5% serum in 0.2% PBS‐T for 45 minutes at RT. Primary antibodies (anti‐MPO, #AF3667, 1:100; anti‐CitH3, #ab5103, 1:1000; anti‐ELA2, #MAB91671, 1:250; anti‐CD34, #M7165, 1:50) were applied overnight at 4°C. After washing, sections were incubated with secondary antibodies (AF 647 donkey anti‐mouse, 1:250, #A‐31571; Invitrogen; AF 488 donkey anti‐rabbit, 1:250, #AF21206; Thermo Fisher; AF555 donkey anti‐goat, 1:250, #A21432; Invitrogen) for 3 hours at RT. Nuclei were counterstained with DAPI (Vector TrueVIEW Autofluorescence Quenching Kit; #SP‐8400‐15).

### Hematoxylin–Eosin Staining of Postmortem Brain Tissue

Deparaffinized and rehydrated sections were first stained with hematoxylin solution for 5 minutes, cleared and differentiated in H_2_O containing 0.2% HCl, rinsed in 70% EtOH, and finally stained with eosin solution for 2 minutes. Sections were then dehydrated and mounted (#1408m; Sakura).

### Imaging

Immunofluorescence images represent maximum intensity projections of z‐stacks that were acquired on an Olympus FV3000 (Evident) confocal microscope with a 60× UPlanApoHR oil objective (NA 1.5), and were post‐processed with Fiji software. Hematoxylin–eosin‐stained slides were scanned using a NanoZoomer automatic digital slide scanner (Hamamatsu Photonics).

### Statistical Analysis

Statistical analyses were carried out using GraphPad Prism (v9.4.1; GraphPad Software) and R (v4.3.1, 4.4; R Foundation for Statistical Computing). Primary analyses were based on within‐subject comparisons between matched samples from the same individual, thereby minimizing the impact of baseline confounders. Where external control data were available, unpaired comparisons were carried out as shown in the text. Normality was assessed using the Shapiro–Wilk test. Paired comparisons were carried out using a paired Student's *t* test for parametric data, and a Wilcoxon matched‐pairs signed‐rank test for nonparametric data, as appropriate. Unpaired comparisons were carried out using an unpaired Student's *t* test for parametric data, and a Mann–Whitney *U* test for nonparametric data. Multiple group comparisons were analyzed using mixed‐effects models to account for missing observations, with Tukey's post hoc correction, or a nonparametric Friedman test followed by Dunn's multiple comparisons test, as appropriate. For analyses involving multiple cytokines, the Bonferroni correction was applied to account for multiple testing. Nonparametric data are reported as the median (interquartile range), whereas parametric data are presented as mean ± SD. Associations between cytokines and clinical stroke parameters were evaluated using generalized linear regression with a gamma distribution and log link. A two‐sided *p* < 0.05 was considered statistically significant.

## Results

### Ischemia Induces Recruitment of Activated Proinflammatory Blood Monocytes

**FIGURE 2:**
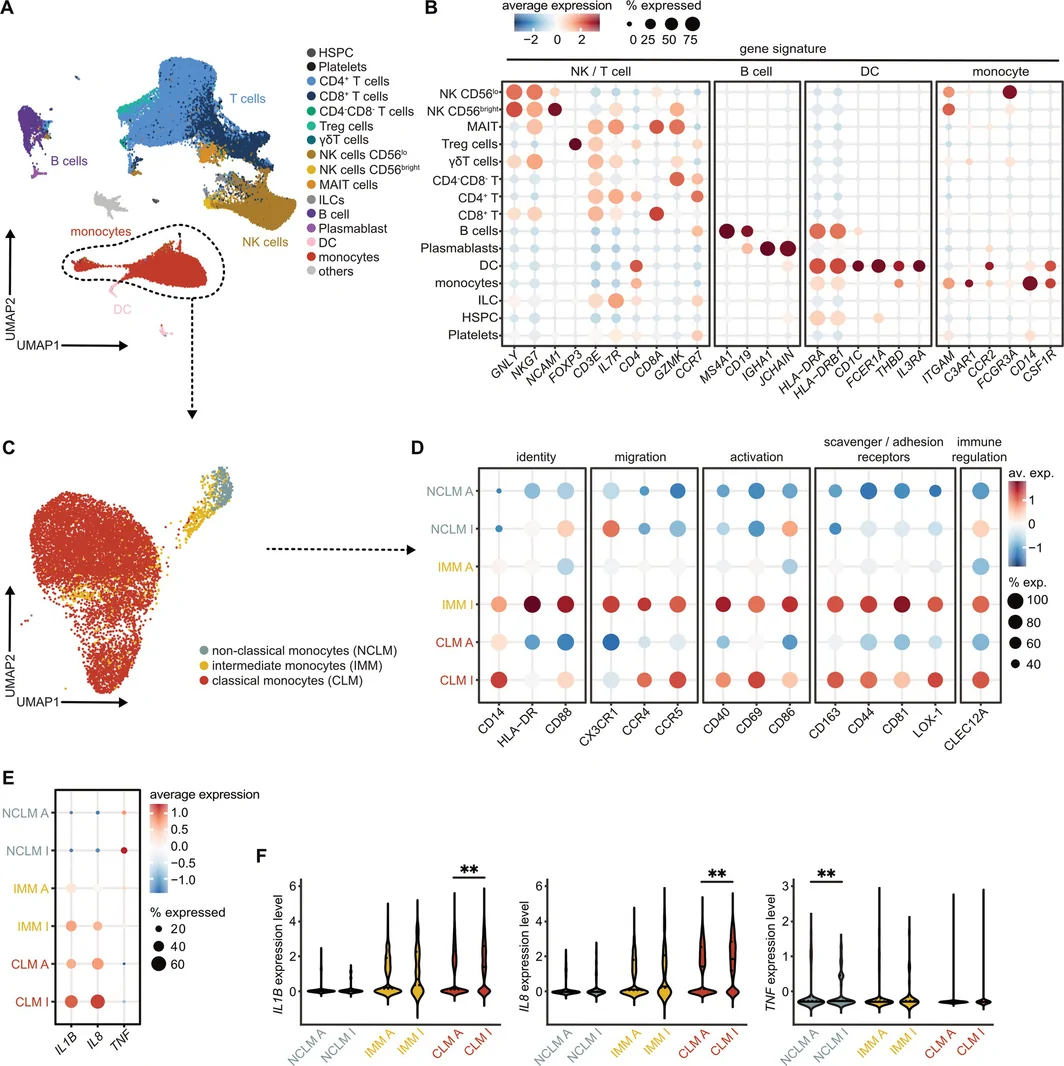
Monocytes from the ischemic blood display an activated phenotype. Peripheral blood mononuclear cells from the ischemic [I] blood were compared with patient‐matched nonischemic arterial [A] blood peripheral blood mononuclear cells (n = 3) using cellular indexing of transcriptomes and epitopes by single‐cell RNA sequencing analysis. (A) UMAP plot of peripheral blood mononuclear cells showing the clusters of major cell populations (color‐coded) identified in ischemic and nonischemic arterial blood. (B) Expression of signature genes used for cell type annotation of the clusters shown in (A). (C) Subclustering of monocytes extracted from the UMAP in (A), showing clusters annotated as CD14^+^CD16^−^ classical (CLM), CD14^+^CD16^+^ intermediate (IMM), and CD14^−^CD16^+^ nonclassical (NCLM) monocytes. (D) Dot plot showing the expression of cell surface protein markers on monocyte subsets from ischemic and nonischemic arterial blood. (E) Dot plot and (F) violin plot showing the RNA expression level of selected cytokines in NCLM, IMM, and CLM from ischemic versus nonischemic arterial blood samples. The *p*‐values were determined using a Wilcoxon matched‐pairs signed‐rank test (**p < 0.01; F). [Color figure can be viewed at www.annalsofneurology.org]

To characterize the hyperacute local immune response in the ischemic brain of stroke patients, we collected ischemic blood samples from the occluded part of the middle cerebral artery during endovascular mechanical thrombectomy in stroke patients with acute LVO (Fig 1). These samples (n = 3) were compared with matched nonischemic carotid artery blood from the same patients using cellular indexing of transcriptomes and epitopes by single‐cell RNA sequencing (Fig 2, and Supplementary Figs S1 and S2). After preprocessing and normalization, as described in the Supplementary Methods, datasets were further analyzed for proinflammatory immune signatures. Single‐cell RNA sequencing analyses revealed the complex immune composition of the ischemic and arterial blood, with several immune and nonimmune cell clusters present (Fig 2A). Clusters were assigned to B‐cell, T‐cell, natural killer cell, and myeloid lineages based on lineage‐specific gene signatures. (Fig 2A,B and Supplementary Fig S2A–C). Monocytes made up one of the more prominent clusters, and were classified based on *ITGAM*, *C3AR1*, *CD14*, *FCGR3A*, and *CSF1R* expression into CD14^−^CD16^+^ nonclassical, CD14^+^CD16^+^ intermediate, and CD14^+^CD16^−^ classical monocytes (Fig 2B,C). Identity was confirmed by surface expression of CD14, HLA‐DR, and CD88, which distinguishes monocytes from dendritic cells (Fig 2D and Supplementary Fig S2D–F).^24^

We investigated the phenotype of these distinct monocyte subsets in ischemic versus nonischemic blood based on surface protein expression in the cellular indexing of transcriptomes and epitopes dataset and on the single‐cell RNA sequencing dataset. Ischemia was associated with a migratory phenotype across all monocyte subsets, as indicated by increased expression of CX3CR1, CCR4, and CCR5 (Fig 2D).^25^ This was accompanied by upregulation of scavenger and adhesion receptors (CD163, CD81, LOX‐1), the immune regulatory receptor CLEC12A, activation markers (CD40 and CD69) on classical and intermediate monocytes, and CD86 across all monocyte subsets in ischemic, but not arterial, blood (Fig 2D). Notably, engagement of CD40 and CD86 are known to be involved in activating intracellular signaling pathways that promote the secretion of proinflammatory cytokines, including IL‐1β^26^ and IL‐8.^27^

Next, we examined whether ischemia induced cytokine expression in blood monocyte subsets. Transcripts for *IL1B* and *IL8* were modestly upregulated in intermediate monocytes, and significantly upregulated in classical monocytes, but not nonclassical monocytes, underscoring the proinflammatory role of classical monocytes during the early immune response to ischemic stroke (Fig 2E,F and Supplementary Fig S1G). In contrast, *TNF* expression was restricted to ischemic blood nonclassical monocytes (Fig 2E,F). Together, these findings identify a distinct activated phenotype of monocytes after ischemic stroke.

### IL‐1β Is Upregulated in the Ischemic Blood Behind the Thrombus

**FIGURE 3:**
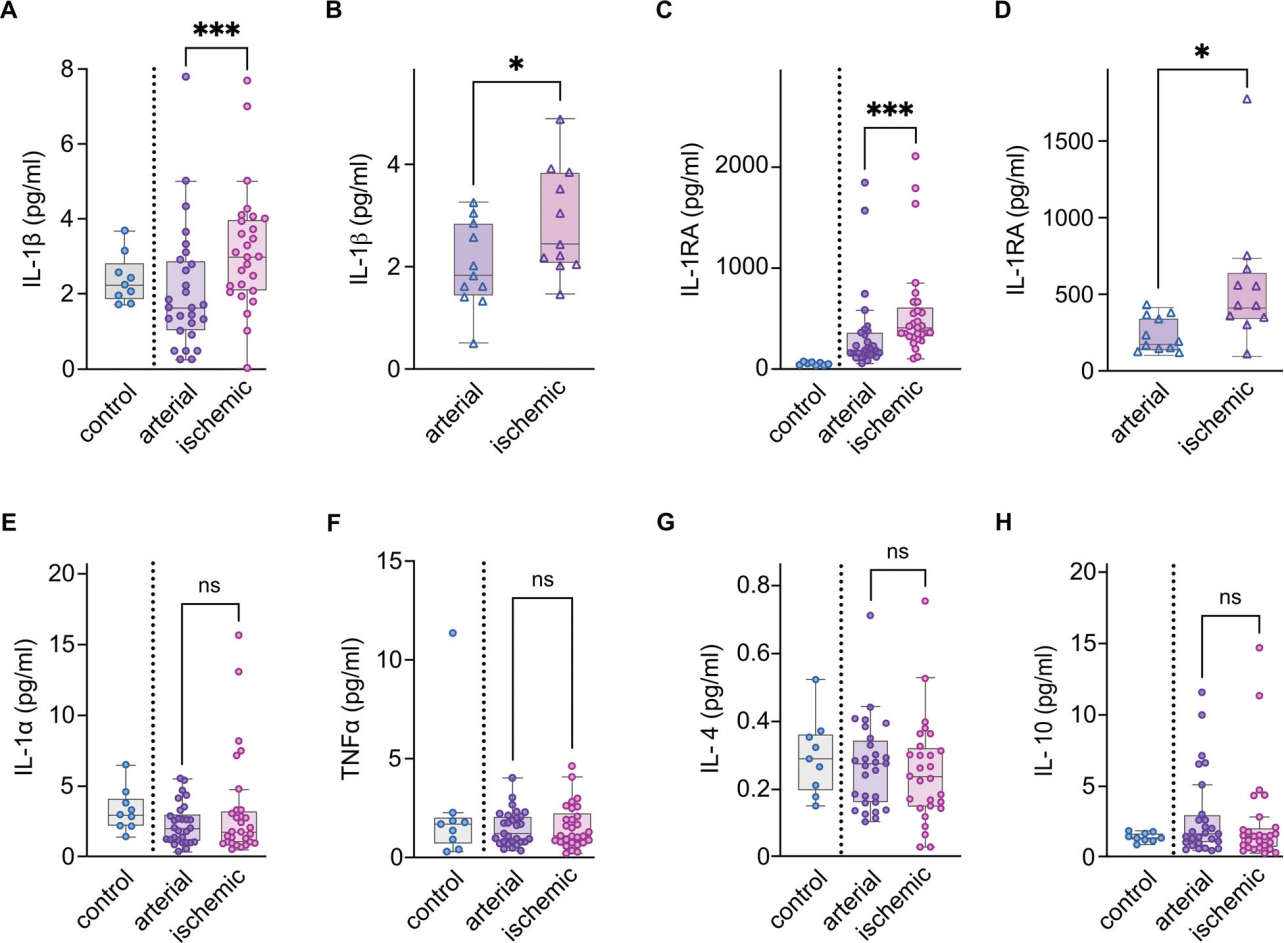
Interleukin (IL)‐1β levels are elevated in the ischemic plasma. Cytokine levels were quantified by electrochemiluminescence immunoassay in the ischemic plasma and compared with matched nonischemic arterial plasma from the same patients (n = 29). Protein expression of (A, B) IL‐1β (B: subgroup <4.5 hours ischemia time, n = 11), (C, D) IL‐1 receptor antagonist (IL‐1RA) (D: subgroup <4.5 hours ischemia time, n = 11), (E) IL‐1α, (F) tumor necrosis factor (TNF)α, (G) IL‐4, and (H) IL‐10 are shown. Additionally, cytokine levels in arterial blood samples from an independent group of nonstroke controls served as a reference (n = 9; separated by a vertical dashed line). Statistical analyses were carried out using the Wilcoxon signed‐rank test for paired comparisons between ischemic and nonischemic arterial samples, with the Bonferroni correction applied to account for multiple testing across cytokines. Missing values: A: n = 2, E: n = 1, F: n = 2. Boxes show the interquartile range with median; whiskers follow the Tukey method. *p < 0.05, ***p < 0.001, ns = not significant. [Color figure can be viewed at www.annalsofneurology.org]

Protein levels of pro‐ and anti‐inflammatory cytokines were subsequently assessed by electrochemiluminescence assays. Matched arterial plasma samples from the ischemic compartment distal to the thrombus and from the nonischemic carotid artery of the same patients were analyzed (n = 29; Fig 3), with arterial blood samples from nonstroke controls serving as the reference (n = 9). In line with our transcriptomic data, IL‐1β levels were significantly higher in ischemic compared with nonischemic arterial samples (2.92 [2.03–3.92] pg/ml vs 1.59 [0.99–2.84] pg/ml; *p* = 0.0006; Fig 3A). No sex‐dependent differences were observed (Supplementary Fig S3A). Subgroup analysis of samples obtained within 4.5 hours after stroke onset demonstrated that this difference was already present in hyperacute stages (Fig 3B). Notably, intravascular IL‐1β increased independent of recombinant tissue plasminogen activator treatment (Supplementary Fig S4A), indicating that early inflammasome‐associated signaling occurs irrespective of thrombolytic therapy, which is known to exert pleiotropic immune effects.^28^ Alongside the upregulation of IL‐1β, we observed higher levels of IL‐1RA (404.7 [315.5–614.4] pg/ml vs 187.4 [137.5–364.8] pg/ml, *p* = 0.0004; Fig 3C), which is a natural competitive inhibitor of IL‐1 signaling with protective effects in experimental stroke.^29^ A significant increase was detectable already within the 4.5‐hour time window (Fig 3D). The concurrent elevation of IL‐1RA indicates simultaneous activation of regulatory components within the IL‐1 signaling pathway. No differences were found for IL‐1α, TNFα, IL‐4, and IL‐10 (Fig 3E–H). Taken together, intravascular blood samples suggested hyperacute inflammasome activation in the ischemic hemisphere.

### Inflammasome Activation in Monocytes Contributes to IL‐1β Production

**FIGURE 4:**
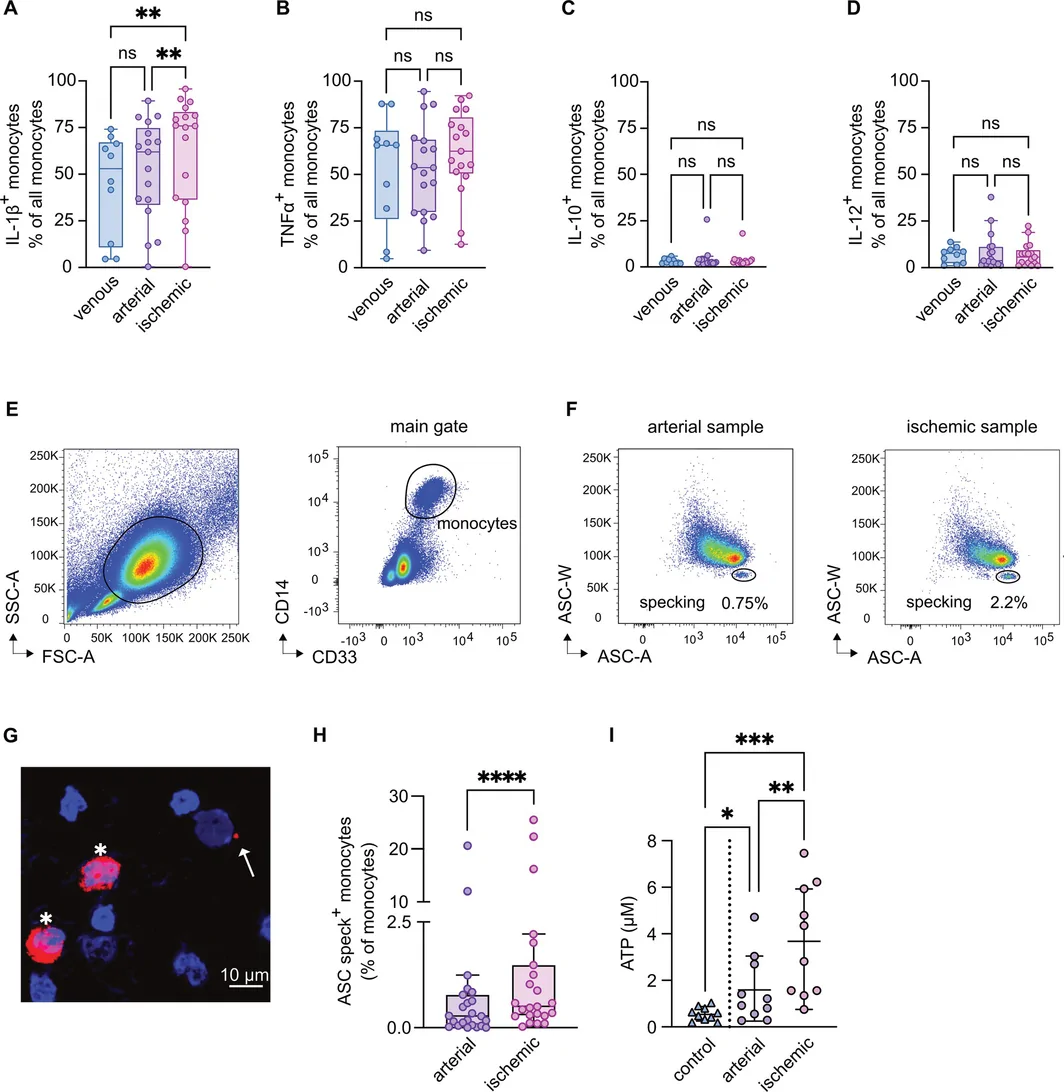
Inflammasome priming in monocytes from the ischemic vasculature. (A–D) Intracellular cytokine levels in monocytes from the occluded segment of the middle cerebral artery (n = 17) were assessed by flow cytometry and compared with matched nonischemic arterial (n = 17) and venous samples (n = 10) after restimulation with 100 ng/ml interferon‐γ and 1.25 μg/ml lipopolysaccharide. (E) Gating strategy to identify apoptosis‐associated speck‐like protein containing a CARD (ASC) speck formation in monocytes. (F) ASC specking is reflected by a reduced pulse width‐to‐area ratio in ischemic and nonischemic arterial samples. (G) Exemplary immunofluorescence image from monocytes with (arrow) and without (*) ASC speck formation (n = 3). (H) Percentage of ASC specking in 2.5 mmol/l adenosine triphosphate (ATP)‐restimulated monocytes from ischemic versus nonischemic arterial blood (n = 23). (I) Liquid chromatography–tandem mass spectrometry‐based analysis of blood ATP levels in matched blood samples (ischemic vs nonischemic arterial; n = 10) and in arterial samples of nonstroke controls (n = 10). Data are shown as median (interquartile range; A–D, H) or mean ± SD (I). Boxes show the interquartile range with the median; whiskers are defined by the Tukey method. *p < 0.05, **p < 0.01, ***p < 0.001, ****p < 0.0001, ns = not significant. Paired group comparisons were carried out by mixed‐effects model followed by Tukey's post hoc correction (A–D). Pairwise comparisons (ischemic vs arterial) were conducted using the Wilcoxon matched‐pairs signed‐rank test (H) or the paired Student's *t* test (I). Unpaired comparisons (control group; separated by a vertical dashed line) were performed using a two‐tailed unpaired Student's *t* test (I). [Color figure can be viewed at www.annalsofneurology.org]

Intracellular IL‐1β expression in monocytes from ischemic and matched nonischemic arterial blood was assessed by flow cytometry after restimulation with interferon‐γ and lipopolysaccharide (n = 17), including additional matched venous samples. IL‐1β levels were significantly higher in monocytes from the ischemic blood compared with matched nonischemic arterial and venous blood (75.8 [36.15–83.45]% vs 61.9 [33.30–74.90]% and 52.9 [10.51–67.23]%; *p* = 0.002 and *p* = 0.006, respectively; Fig 4A and Supplementary Fig S5). In contrast, intracellular TNFα, IL‐10, and IL‐12 remained unchanged, indicating selective early IL‐1β induction (Fig 4B–D).

An antibody detecting both pro‐ and mature IL‐1β was used, with elevated intracellular levels indicating inflammasome priming.^30^ To further assess priming, ASC speck formation was analyzed by flow cytometry following ATP licensing (Fig 4E,F).^31^, ^32^ Monocytes from ischemic blood showed a higher proportion of ASC speck‐positive cells compared with matched nonischemic arterial samples (0.50 [0.28–1.48]% vs. 0.27 [0.048–0.78]% *p* < 0.0001; Fig 4G,H).

After priming, full inflammasome activation requires additional licensing cues. ATP is a danger signal capable of activating the inflammasome.^33^ In cerebral ischemia, ATP is rapidly released into the extracellular space with detrimental effects on stroke outcome.^34^ To assess intravascular ATP levels, blood samples were collected into nucleotidase inhibitor‐containing tubes to prevent ATP degradation. Analysis of ATP plasma levels by liquid chromatography–tandem mass spectrometry showed significantly higher levels of ATP in the ischemic compared with matched nonischemic arterial blood (n = 10; 3.68 ± 2.39 μmol/l vs 1.59 ± 1.45 μmol/l, *p* = 0.002; Fig 4I). Notably, ATP levels in both ischemic and nonischemic samples were also elevated relative to arterial plasma from nonstroke controls (n = 10; 0.56 ± 0.29 μmol/l, *p* = 0.0007 and *p* = 0.04, respectively), confirming the surge in ATP levels observed after stroke in animal models.^34^ Taken together, our findings indicate the presence of monocyte inflammasome priming alongside elevated ATP levels in ischemic plasma, consistent with conditions permissive for inflammasome activation within the ischemic vasculature.

### Neutrophil Activation in the Ischemic Blood Compartment

**FIGURE 5:**
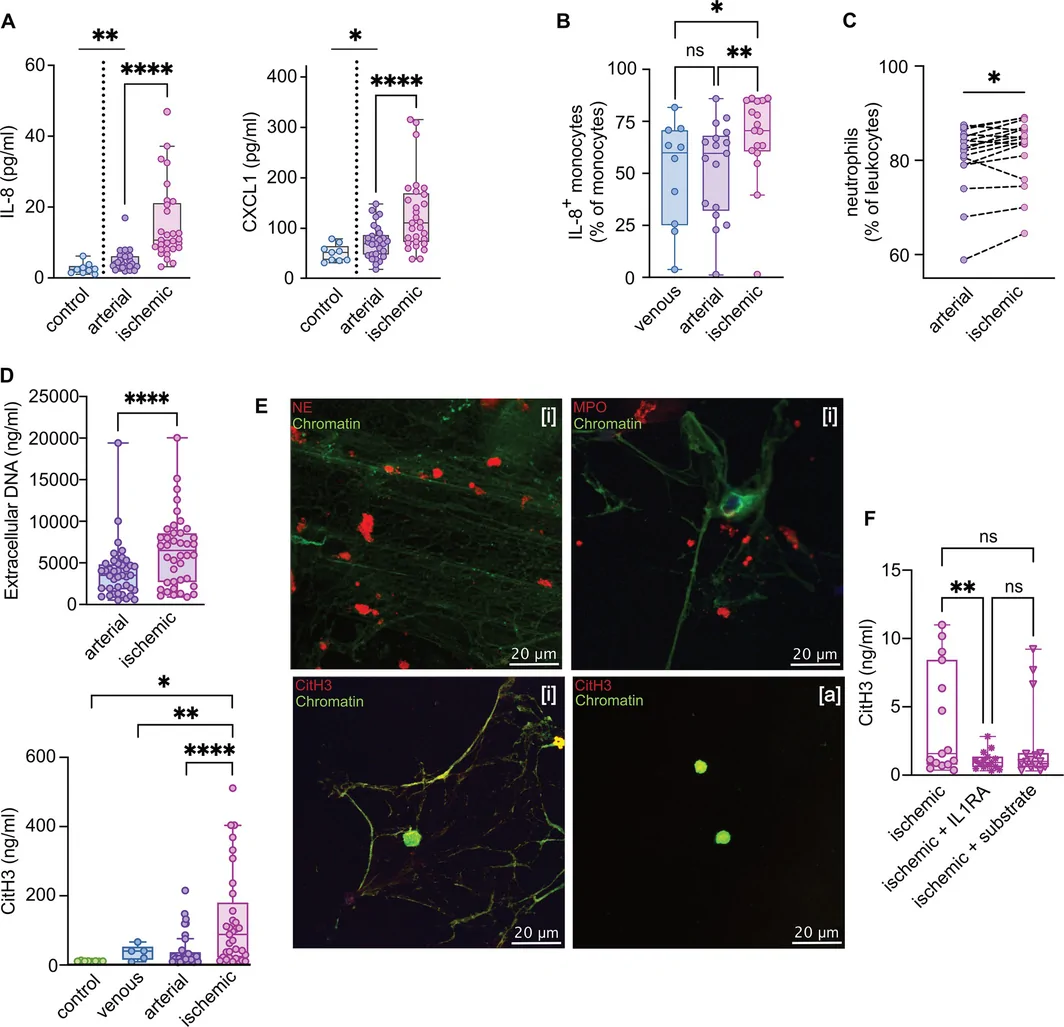
Activated neutrophils accumulate in the ischemic intravascular compartment behind the thrombus. (A) Analysis of plasma neutrophil chemoattractants interleukin (IL)‐8 and C‐X‐C motif chemokine ligand 1 (CXCL1) in matched ischemic and nonischemic arterial (n = 29), as well as nonstroke arterial control samples (separated by a vertical dashed line, n = 9) using electrochemiluminescence immunoassays. (B) Intracellular IL‐8 expression in monocytes analyzed by flow cytometry following restimulation with 100 ng/ml interferon‐γ and 1.25 μg/ml lipopolysaccharide (n = 17). (C) Relative neutrophil count, shown as percentage of total leukocytes, as assessed by flow cytometry in paired ischemic and arterial control samples (n = 17). (D) Blood neutrophil extracellular trap (NET) formation quantified by extracellular DNA and citrullinated histone H3 (CitH3) levels. Extracellular DNA was analyzed in ischemic and matched nonischemic arterial plasma (n = 40). CitH3 was measured in ischemic versus matched nonischemic arterial (n = 33) and venous (n = 5), as well as nonstroke arterial control plasma (n = 8). (E) Ischemic plasma [i] shows NET‐inducing properties ex vivo, when added as a stimulant to the culture medium of healthy donor neutrophils (n = 3). Immunofluorescence imaging showing distinct patterns of extracellular chromatin (green) and neutrophil elastase (NE), myeloperoxidase (MPO), and CitH3 (red), respectively. No NET formation was observed when stimulating equally with nonischemic arterial control blood [a] (n = 3). (F) CitH3 levels in the supernatant of cultured neutrophils from healthy donors after stimulation with 10% ischemic arterial blood for 4 h (n = 15). IL‐1 receptor antagonist (IL‐1RA) or control substrate were added simultaneously to neutralize IL‐1β. Statistical analysis: Paired comparisons between ischemic and matched nonischemic arterial samples were performed using a Wilcoxon matched‐pairs signed‐rank test with the Bonferroni correction. Unpaired comparisons between nonischemic arterial samples from stroke patients and arterial samples from nonstroke controls were carried out using a Mann–Whitney *U* test (A). Intracellular cytokine levels across blood compartments were analyzed using mixed‐effects models with Tukey's post hoc correction (B). Paired neutrophil counts were analyzed using the Wilcoxon signed‐rank test (C). NET‐associated markers were analyzed using the Wilcoxon signed‐rank test or mixed‐effects models with Tukey's post hoc correction, as appropriate (D). Ex vivo NET stimulation experiments were analyzed using a Friedman test followed by Dunn's multiple comparisons correction (F). Data shown as median (interquartile range). *p < 0.05, **p < 0.01, ****p < 0.0001, ns = not significant. [Color figure can be viewed at www.annalsofneurology.org]

IL‐1β‐associated signaling has been linked to the regulation of neutrophil chemoattractants, such as CXCL1 and CXCL8 (IL‐8).^7^, ^35^ Plasma concentrations of CXCL1 and IL‐8 were therefore quantified by targeted proteomics. Both IL‐8 (10.95 [8.42–21.55] pg/ml vs 4.18 [3.11–6.37] pg/ml; *p* < 0.0001) and CXCL1 (110.9 [72.10–170.4] pg/ml vs 69.55 [47.02–87.57] pg/ml; *p* < 0.0001) were significantly increased in ischemic compared with matched nonischemic arterial plasma (Fig 5A). Levels were also higher in nonischemic arterial samples from stroke patients than in nonstroke controls (IL‐8: 2.59 [1.66–3.57] pg/ml, *p* = 0.006 and CXCL1: 52.19 [36.52–65.49] pg/ml *p* = 0.049), indicating a chemotactic gradient toward the ischemic brain.

Chemokines can be produced by both immune and nonimmune cells. To assess monocyte‐associated chemokine expression, intracellular IL‐8 levels were analyzed by flow cytometry after restimulation with interferon‐γ and lipopolysaccharide. The proportion of IL‐8‐positive monocytes was higher in ischemic blood samples compared with matched nonischemic arterial and venous samples (71.0 [60.80–85.45]% vs. 60.0 [32.00–68.80]% and 60.25 [25.00–71.48]%; *p* = 0.002 and *p* = 0.021, respectively; Fig 5B). To assess neutrophil abundance across compartments, neutrophils were quantified in matched blood samples. A higher proportion of neutrophils was observed in ischemic compared with nonischemic arterial blood samples (n = 17, *p* = 0.012; Fig 5C).

Upon activation, neutrophils release NETs, which are highly prothrombotic and toxic to surrounding cells.^11^, ^36^ NET‐associated markers were analyzed to assess intravascular NET formation in hyperacute stroke. Levels of extracellular DNA were significantly higher in ischemic compared with matched nonischemic arterial plasma (6,515 [2,655–8,618] ng/ml vs 3,501 [1,780–4,858] ng/ml; *p* < 0.0001). In addition, concentrations of CitH3,^37^ a marker of NETosis, were significantly increased in ischemic samples compared with matched nonischemic arterial (81.5 [14.45–175.4] ng/ml vs 13.0 [3.05–30.65] ng/ml; *p* < 0.0001) and venous samples (33.3 [7.40–46.60] ng/ml; *p* = 0.005), as well as arterial samples from nonstroke controls (3.2 [2.50–3.85] ng/ml; *p* = 0.020; Fig 5D). No significant sex‐dependent differences were observed for IL‐8, CXCL1, CitH3, or extracellular DNA (Supplementary Fig S3B–E). Notably, when analyzing plasma samples obtained within 4.5 hours after ischemia onset, we observed that NET markers were already elevated during the hyperacute phase and were independent of recombinant tissue plasminogen activator treatment (Supplementary Figs S4B and S6).

To assess whether ischemic plasma contains factors associated with NET induction ex vivo, neutrophils from healthy donors were incubated with ischemic plasma and matched nonischemic arterial plasma from stroke patients (n = 3), and NET formation was analyzed by confocal microscopy. NET formation was observed after stimulation with ischemic plasma, whereas matched nonischemic arterial plasma did not induce detectable NET formation (Fig 5E). To examine whether IL‐1β present in ischemic plasma is associated with NET formation, ischemic plasma samples were incubated with IL‐1RA (n = 15). CitH3 levels were lower after IL‐1β antagonism (*p* = 0.004) compared with an inactive control substrate (Fig 5F), suggesting an association between IL‐1β signaling and NET formation in this ex vivo setting.

Together, these findings show that ischemia rapidly establishes a chemokine‐rich intravascular milieu that drives neutrophil accumulation and promotes early NET formation, partly through IL‐1β signaling.

### NET Formation in Human Ischemic Brain Tissue After Acute Stroke

**FIGURE 6:**
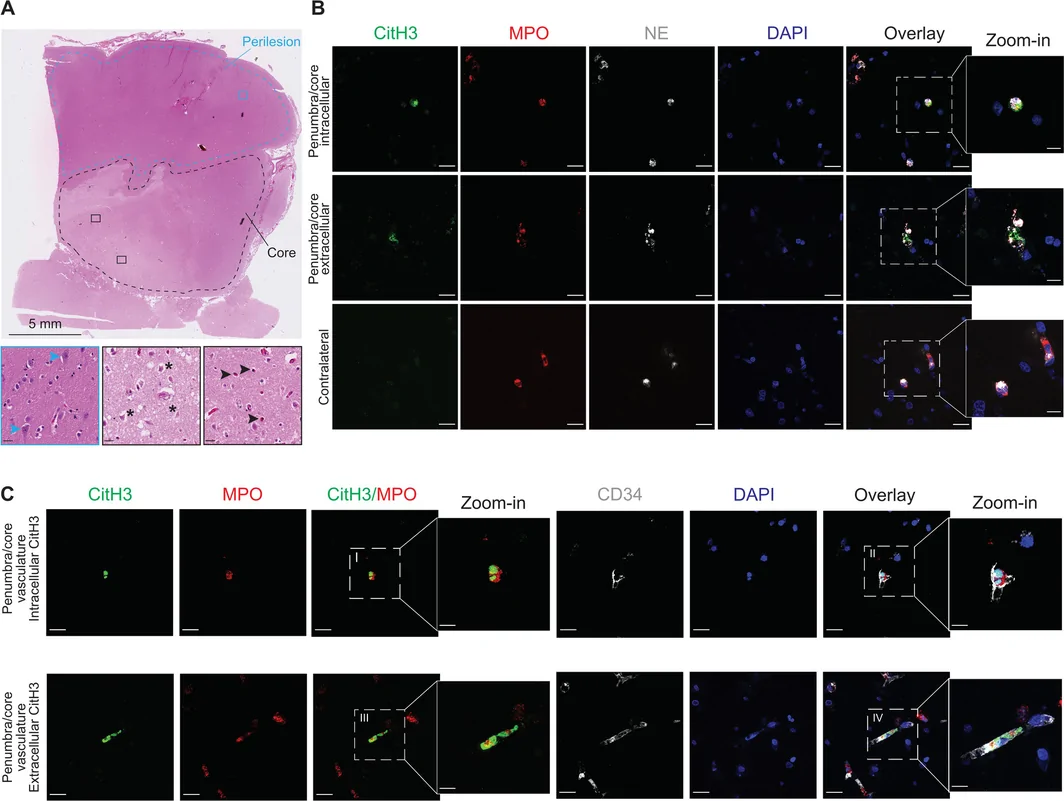
Neutrophil extracellular trap formation in human postmortem formalin‐fixed paraffin‐embedded brain tissue from the ischemic penumbra. Tissue was processed 4 days after occlusion of middle cerebral artery (n = 3). (A) Hematoxylin–eosin staining of postmortem brain tissue illustrating the ischemic core (black dashed outline) and penumbral region (blue dashed outline) with intact neurons visible in the zoom‐in (blue arrows). Ghost cells with perineuronal edema (zoom‐in, asterisks) and eosinophilic neurons with pyknotic nuclei (zoom‐in, black arrows) indicate neurons undergoing ischemic necrotic cell death in the core region (zoom‐in scale bar: 20 μm). (B) Immunofluorescence staining of neutrophil extracellular trap formation (n = 3) identified by colocalization of citrullinated histone H3 (CitH3), myeloperoxidase (MPO), neutrophil elastase (NE), and DAPI (penumbra vs contralateral hemisphere). (C) Further colocalization with the endothelial cell marker CD34 allows distinction between intravascular and extravascular neutrophil extracellular trap formation. [Color figure can be viewed at www.annalsofneurology.org]

To assess NET formation in human ischemic brain tissue in situ, we obtained autoptic brain specimens (n = 3) 4–5 days after middle cerebral artery occlusion. Brain tissue was collected within 8 hours postmortem, ensuring high tissue integrity (Fig 6A). In line with previous reports^11^ and our data from ischemic plasma, we could document NET formation in ischemic brains by the colocalization of CitH3, myeloperoxidase, and neutrophil elastase (Fig 6B,C). NET structures were confined to the ipsilateral ischemic hemisphere and were predominantly localized within the intravascular compartment, with no NETs detected in the contralateral hemisphere.

### Association of Proinflammatory Plasma Markers with Infarct Size and Duration of Ischemia

**FIGURE 7:**
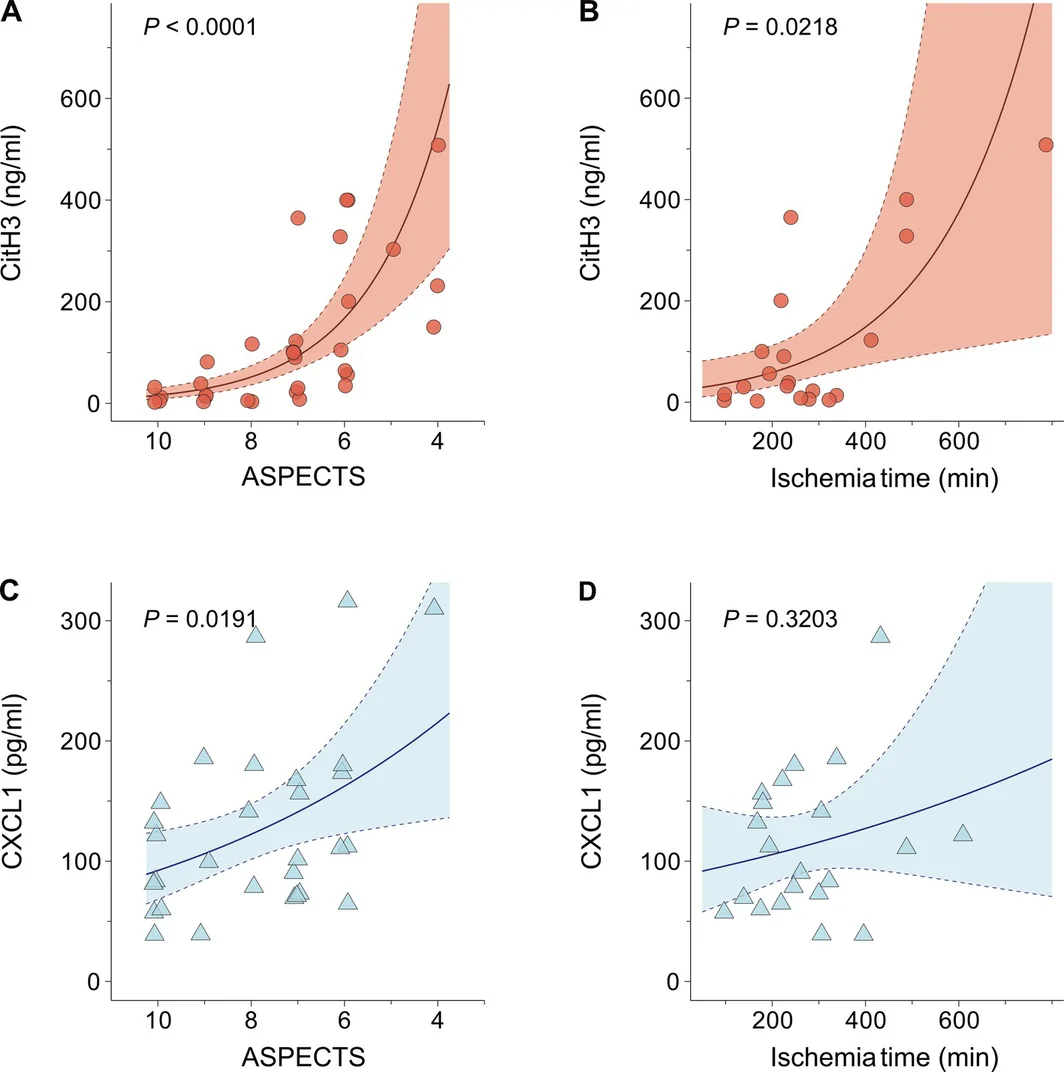
Association of proinflammatory plasma markers with infarct size and duration of ischemia. Correlation between radiological (ASPECTS) and clinical (ischemia time) indicators of stroke severity and serum concentration of citrullinated histone H3 (CitH3) and C‐X‐C motif chemokine ligand 1 (CXCL1), respectively. Point markers represent individual subjects (A and B: n = 33 independent patients, ischemia time missing in 12; C and D: n = 29 independent patients, ischemia time missing in 8). Lines indicate point estimates (solid) and pointwise 95% confidence intervals (dashed) of conditional mean concentrations of CitH3 and CXCL1 obtained from generalized linear regression modeling with a gamma response distribution and logarithmic link function. Inset *p*‐values were obtained using Wald's method. [Color figure can be viewed at www.annalsofneurology.org]

We next assessed the associations between locally increased plasma markers and clinical parameters. Generalized linear regression model with gamma response distribution analysis was used to investigate whether CitH3 (n = 33) or the neutrophil attracting chemokine CXCL1 (n = 29) in the ischemic plasma were associated with stroke size or duration of ischemia. We assessed infarct size using the Alberta Stroke Program Early CT Score (ASPECTS), a quantitative score that evaluates the extent of early ischemic changes. Notably, CitH3 was positively associated with both infarct size and duration of ischemia (*p* < 0.0001 and *p* = 0.022, respectively; Fig 7A,B). Further supporting the role of neutrophils, we found that plasma CXCL1 levels were also significantly associated with infarct size (*p* = 0.019; Fig 7C,D). In contrast, no association was observed between plasma levels of IL‐1β, IL‐1RA, or IL‐8 and infarct extent or ischemia duration (Supplementary Fig S7).

## Discussion

The present study demonstrates that highly conserved sterile immune signaling pathways are activated intravascularly within the ischemic hemisphere within hours of an acute large vessel occlusion. Through direct analysis of the intravascular compartment in the hyperacute phase of human ischemic stroke, our data provide descriptive evidence of early thromboinflammatory signaling consistent with mechanisms described in experimental models.^38^, ^39^, ^40^

In this context, the detection of increased intravascular IL‐1β protein levels indicates full inflammasome activation, including active release of IL‐1β, within the ischemic compartment. Cellular indexing of transcriptomes and epitopes by single‐cell RNA sequencing and flow cytometry analyses showed priming‐related changes in subsets of monocytes, suggesting that intravascular monocytes contribute, at least in part, to local IL‐1β production. These observations are consistent with an ischemic milieu enriched in damage‐associated molecular patterns, as reported in prior studies.^41^, ^42^, ^43^ In addition, we observed markedly elevated extracellular ATP levels, a potent inflammasome‐licensing signal, which, together with damage‐associated molecular patterns, may facilitate full inflammasome activation within the intravascular compartment.^30^, ^44^

In line with observations reported in a recent study by Walle et al, acute IL‐1β release appears to be restricted to a limited fraction of monocytes rather than the entire population. Given the close association between IL‐1β release and pyroptosis, this small subset might serve a sentinel‐like function, initiating inflammatory signaling while preserving the broader monocyte pool. Despite their limited number, such cells have been reported to account for a disproportionate share of IL‐1β production,^45^, ^46^ a concept that aligns with our descriptive findings. Although absolute IL‐1β levels in our study were low compared with pathogen‐driven hyperacute immune responses,^47^ similar concentrations have been associated with adverse outcomes in cardiovascular disease.^48^, ^49^ This supports the notion that low circulating IL‐1β levels do not preclude biologically relevant vascular activity. Functional studies have demonstrated that minimal IL‐1 receptor occupancy is sufficient to elicit robust biological responses,^50^ and that concurrent expression of the IL‐1RA constrains, but does not abolish, IL‐1β signaling.^51^ Accordingly, low intravascular IL‐1β levels may remain biologically active despite IL‐1RA upregulation.

In addition to IL‐1β, the neutrophil chemoattractants, IL‐8 and CXCL1, were significantly increased within the ischemic hemisphere, and were accompanied by a relative increase in neutrophils in the ischemic compartment. A similar neutrophil accumulation has previously been reported by Kollikowski et al and attributed to collateral blood flow.^52^, ^53^ Although elevated IL‐8 or CXCL1 levels were not reported in that study, differences in cytokine detection across studies may partly reflect methodological variability in assay platforms and sensitivity, but are also likely influenced by cohort characteristics, including more extensive ischemic injury, as reflected by lower ASPECT scores, and longer ischemia durations in our patients. Blood monocytes were identified as an IL‐8‐producing cell population, further underscoring their relevance in early intravascular inflammation.

Neutrophils are among the earliest immune cells recruited to the ischemic vasculature and represent key effectors of thromboinflammatory injury.^9^, ^54^, ^55^ Within the ischemic cerebral vasculature, neutrophils are rapidly activated. This notion is supported by Kollikowski et al, who reported platelet–neutrophil interactions within the collateral circulation of ischemic stroke patients, characterized by elevated platelet‐derived CXCL7, a potent inducer of neutrophil degranulation and intravascular activation.^56^ Such platelet‐driven priming might facilitate downstream neutrophil effector responses, including NET formation under ischemic conditions. In line with this, we observed a significant intravascular enrichment of NET‐associated components.

Experimental stroke models have shown NET formation as early as 6 hours after stroke onset with predominant intravascular localization,^36^ and our exemplary histological analyses of autopsy‐derived human stroke tissue similarly point toward preferential intravascular NET formation within ischemic brain regions. Importantly, even in the absence of transmigration into the brain parenchyma, intravascular neutrophil accumulation and activation may already impair downstream microvascular blood flow.^8^, ^57^, ^58^

Damage‐associated molecular patterns have been identified as potent NET inducers,^11^ and IL‐1β has likewise been implicated in NET formation.^59^ In line with this, exploratory ex vivo analyses in our study suggested that IL‐1β antagonism is associated with reduced NET formation, indicating a potential functional link between inflammasome‐related signaling and prothrombotic NET generation.

Given the limited sample size, these observations warrant cautious interpretation and further investigation to clarify underlying mechanisms and potential therapeutic relevance. Previous studies have reported bidirectional interactions between inflammasome activation and NET formation, as NETs themselves can trigger inflammasome activation in myeloid cells.^60^ In this descriptive context, our findings are compatible with an association between inflammasome‐related pathways and NET formation during the hyperacute phase of ischemic stroke, suggesting that these processes may occur concomitantly as components of early thromboinflammatory responses. Notably, neutrophil activation is also influenced by multiple factors independent from ischemia, including circadian rhythmicity and age, contributing to a complex and highly individualized inflammatory landscape.^54^, ^61^

Given the advanced age of our cohort, age‐related dysregulation of granulopoiesis and altered inflammasome responsiveness, collectively referred to as inflammaging, may favor the emergence of prothrombotic neutrophil subsets, which have been implicated in microvascular obstruction and adverse stroke outcomes.^54^ However, neutrophils represent a heterogeneous immune cell population with context‐dependent functions, also exerting reparative effects either directly through the clearance of necrotic cellular debris or indirectly by shaping the local immune milieu.^62^, ^63^ Accordingly, selective targeting of specific effector pathways, such as excessive NET formation or inflammasome‐associated signaling, might be preferable to broad inhibition, which has been associated with adverse clinical outcomes.^64^ Despite a relatively homogeneous cohort with respect to stroke etiology, our dataset revealed marked interindividual variability in inflammatory signatures. Although associations were observed between ischemic injury extent and selected neutrophil activation markers (NETs, CXCL1), no consistent linear relationship was observed across the broader immune marker panel (IL‐1β, IL‐8). Collectively, this heterogeneity suggests that infarct characteristics, such as lesion size, location, and occlusion time, alone may not adequately capture inflammatory activity, and that biomarker‐based stratification could be required to define inflammatory phenotypes in ischemic stroke.

Although limited by its observational design and inability to establish mechanistic causality, this study provides unique insights into human intravascular immune responses during the hyperacute phase of ischemic stroke. Our descriptive data document early activation of thromboinflammatory pathways after large vessel occlusion that are consistent with mechanisms identified in preclinical models. In this clinical context, the SCIL‐Stroke trial showed that IL‐1 receptor antagonism is safe and effectively reduces systemic inflammatory markers in ischemic stroke, although it was not powered to detect effects on clinical outcome.^65^ Importantly, the causal relevance of IL‐1β‐mediated inflammation for vascular events has been established by the CANTOS trial, in which selective IL‐1β inhibition reduced major cardiovascular events.^66^ Together, these observations support further investigation of IL‐1β‐associated thromboinflammatory pathways in ischemic stroke. Recent encouraging studies, including the phase I/IIa APRIL trial of Toll‐like receptor antagonism, show that early immunomodulation in acute ischemic stroke is feasible and safe, and might be associated with positive clinical effects, consistent with the role of Toll‐like receptor 4 as an upstream trigger of inflammasome activation.^67^

In summary, this study provides a detailed descriptive characterization of the hyperacute intravascular immune response in ischemic stroke and reveals marked interindividual heterogeneity. These data point to a relevant intravascularly initiated immune cell activation. To what extent this inflammatory response propagates from the vasculature into the ischemic brain parenchyma, and vice versa, remains to be determined in future studies. Adequately powered clinical trials will be required to assess whether targeted immunomodulatory strategies targeting these pathways, potentially guided by biomarker‐based patient stratification, can translate into improved clinical outcomes.

## Author Contributions

M.G. and J.M. contributed to the conception and design of the study. J.M., M.G., H.E., M.O., C.C., B.R., A.W., J.H., E.S., B.C., A.J., K.D., C.B., M.P., I.S.S., M.Z., H.C., L.W., M.B., F.F., U.H., B.H., J.F., T.F., R.H., B.H.C., R.D., G.T., E.T., I.P., and T.V.A. contributed to the acquisition and analysis of data. J.M., M.G., B.R., T.M., E.T., T.R., and A.M.S. contributed to drafting the manuscript or preparing the figures.

## Potential Conflicts of Interest

Nothing to report.

## Supporting information


**Table S1** Demographics and characteristics of the non‐stroke control group.
**Table S2**. Flow cytometry antibodies.
**Table S3**. ATP inhibitory mixture.
**Figure S1**. Single‐cell quality control plots in arterial and ischemic blood samples. (A) Violin plots displaying nFeature RNA, nCounts RNA, the percentage of mitochondrial genes (percent.mt), the percentage of ribosomal genes (percent.ribo) and the percentage of heat shock protein genes (percent.hs). (B) Bar plots summarizing the distribution of major cell populations across conditions. (C) UMAP projection based on RNA‐derived integration embeddings.
**Figure S2**. Immune cell profiling of arterial and ischemic blood. (A) UMAP plot showing clusters and annotation of B cells identified in ischemic and non‐ischemic arterial blood. (B) UMAP plot showing clusters and annotation of T cells identified in ischemic and non‐ischemic arterial blood. (C) UMAP plot showing clusters and annotation of NK cells identified in ischemic and non‐ischemic arterial blood. (D) Dot plot showing the RNA expression levels and frequency of cell surface protein markers within NCLM, IMM, and CLM from arterial versus ischemic blood samples. (E) Dot plot and (F) violin plots showing the expression levels of CD88 on annotated immune cell populations and on NCLM, IMM, and CLM from arterial versus ischemic blood samples (including median and interquartile range). (G) Density UMAP analyses showing the protein expression levels of cytokines by NCLM, IMM, and CLM from arterial versus ischemic blood samples. *P*‐values were determined using a Wilcoxon matched‐pairs signed‐rank test (**p < 0.01).
**Figure S3**. Inflammatory immune mediator plasma levels stratified by sex in ischemic blood samples. Plasma concentrations of inflammatory mediators (A) IL‐1*β* (n = 27), (B) IL‐8 (n = 29), (C) CXCL1 (n = 29), (D) CitH3 (n = 33) and (E) extracellular DNA (exDNA; n = 40) are shown for men and women. Data are shown as median (IQR); n.s. = not significant. Statistical analysis was performed using the Mann–Whitney U test for unpaired samples.
**Figure S4**. Inflammatory immune mediator plasma levels in patients with and without rtPA treatment. Plasma concentrations of inflammatory mediators (A) IL‐1*β* (n = 27) and (B) CitH3 (n = 33) were quantified in acute ischemic stroke patients stratified by intravenous rtPA treatment. Data are shown as median (IQR). **p < 0.01, ***p < 0.001, and ****p < 0.0001. Statistical analysis was performed using the non‐parametric Wilcoxon signed‐rank test for paired comparisons.
**Figure S5**. Intracellular cytokine expression in monocytes. Exemplary FACS plots displaying varying cytokine expression of IL‐1*β*, IL‐8, TNF*α*, IL‐10 and IL‐12 after restimulation with 100 ng/ml IFN*γ* and 1.25 μg/ml LPS in (A) ischemic, (B) matched non‐ischemic arterial and (C) venous blood samples from one stroke patient.
**Figure S6**. NET markers are already elevated within the first 4.5 hours after stroke onset. CitH3 (n = 12) levels in the ischemic blood plasma compared to matched non‐ischemic arterial plasma with an overall maximum ischemia time of 4.5 hours. Statistical analysis was performed using the non‐parametric Wilcoxon signed‐rank test for paired comparisons. Data shown as median (IQR). ***p < 0.001.
**Figure S7**. Association between stroke severity and serum biomarkers. Correlation between ASPECTS and ischemia duration and the serum concentrations of (A, B) IL‐1*β* (n = 27), (C, D) IL‐1RA (n = 29), and (E, F) IL‐8 (n = 29). A linear regression model was used for statistical analysis. The light blue shaded area represents the 95% confidence interval. Ischemia time was missing for (B) n = 7, (D) n = 8, and (F) n = 8.

## Data Availability

Single‐cell sequencing data are available at NCBI Gene Expression Omnibus (GEO; #GSE285659). Further data from sample analysis will be made available on reasonable request to the corresponding author.
