# Supplementary material for: Hyperacute Interleukin‐1β Production and Neutrophil Extracellular Trap Formation in the Cerebral Circulation of Stroke Patients with Large Vessel Occlusion
Source: Ann Neurol. 2026 Jun 22;100(4):824–40. doi: 10.1002/ana.78284 (PMC13587107; doi:10.1002/ana.78284)
Supplement: Supplementary file 1 [file ANA-100-824-s001.docx]

**Supplementary Methods**

**Study inclusion and exclusion criteria**

Patient inclusion criteria were defined as follows:

(I) Acute ischemic stroke caused by LVO of the proximal middle cerebral artery (M1- or M2-segment) confirmed by multimodal CT imaging or magnetic resonance imaging. (II) Patients must be eligible for a thrombectomy procedure by standard care guidelines (extended therapeutic time window ≤ 24 hours).

Patient exclusion criteria were defined as follows:

(I) Confirmed LVO other than proximal MCA-occlusion. (II) Angiographically proven restored antegrade blood flow before collection. (III) Blood samples which could not be processed within 6 hours of sample acquisition. (IV) Patients with known autoimmune disease. (V) Patients with relevant acute infections, defined by elevated markers of systemic infection, proof of urinary tract infection, pneumonia or endocarditis. (VI) Patients with known malignancies.

**Sample acquisition protocol**

Mechanical thrombectomies were performed by experienced interventional neuroradiologists on a biplane angiography system. Endovascular access was achieved via a transfemoral approach through the common femoral artery using the Seldinger technique with placement of a 9F-sheath. After confirmation of the intracranial occlusion, a guide catheter was advanced into the internal carotid artery (ICA). Subsequently the intracranial occlusion was passed with a microcatheter (Headway 21, Microvention Terumo) equipped with a microwire (Traxcess 0.014”, Microvention Terumo). After the successful placement of the microcatheter distal to the occlusion, gentle aspiration with a 5 ml syringe was performed (Figure 1). After the refusal of the first 1 ml of blood, another 0.5 -1.0 ml was aspirated for laboratory analysis (ischemic arterial sample). Then mechanical thrombectomy was immediately continued. At the end of the intervention further blood samples were collected first from the cervical ICA or aorta (non-ischemic arterial sample) and second from peripheral venous access (venous sample). Blood samples were immediately placed on a rocker and sent to the laboratory as soon as possible.

**PBMC isolation**

Blood (1:3 diluted) was layered on Ficoll-Paque^TM^ Plus (#17144002, cytiva) and centrifuged for 25 min at 690 g with break off at RT. The mononuclear cell-rich layer was collected.

**Single-cell sequencing data processing**

Sequence reads were aligned to the reference human genome GRCh38, following the generation of barcode-gene matrices via Cell Ranger v6.0.2 (10x Genomics). We excluded cells with <800 and >20000 genes, <500 features and >10 reads mapping to the mitochondrial genome. Doublets were identified and removed using DoubletFinder (version 2.0.4). Single-cell sequencing data are available at NCBI Gene Expression Omnibus (GEO; #GSE285659).

**Computational analysis (Seurat)**

Using Seurat (version 5.0.3), counts were log-normalized for each cell using the natural logarithm of 1 + counts per ten thousand. The 2,000 most variable genes were identified by using FindVariableFeatures function. We standardized expression values for each gene across all cells by standard-score transformation (ScaleData). Principal component analysis (PCA) was performed on the scaled variable gene matrix. To account for batch effects, we normalized each sample and filtered based on the above quality control metrics independently before merging the count matrices into a single matrix and integrating samples via Seurat with the function IntegrateLayers and the method CCAIntegration. We performed PCA analysis in Seurat using the gene expression data (50 PCs) followed by dimensional reduction (UMAP, dims 1:20). We calculated nearest neighbors and performed Louvain clustering in Seurat (resolution = 0.8).

**Plasma cytokine analysis**

Plasma samples were diluted three-fold in Diluent 57 and run in duplicates. Plates were read using the SECTOR Imager 6000 Plate Reader (Mesoscale Discovery), and the data were analysed using the MSD Discovery Workbench software.

**Ex vivo stimulation of whole blood and flow cytometry**

100 µl of whole blood was transferred to FACS tubes within 4 hours after sample collection. Cells were primed with IFNγ (100 ng/ml) for 2 hours, followed by 1.25 μg/ml LPS-restimulation for an additional 6 hours. A Golgi stop solution (#00-4506, eBioscience and #420701, Biolegend) was added after 3 hours. After cell surface staining and red blood cell lysis, permeabilization was performed with 0.3% saponin buffer (#S4521, Sigma-Aldrich), followed by intracellular cytokine staining.

**ASC speck imaging**

For immunofluorescence staining cells were fixed with 4% PFA, permeabilized with 0.2% Tween (#P749, Sigma-Aldrich) and blocked with 10% AB-serum (#126615, Sigma-Aldrich) for 10 minutes at RT. Secondary antibody staining was performed with Alexa Fluor 488 donkey anti-mouse (1:200, #A21202, Invitrogen) and Alexa Fluor 555 donkey anti-rabbit (1:400; #A31572, Invitrogen).

**ATP inhibitory mixture**

For ATP measurement plasma samples were spiked with an inhibitory mixture (Supplementary Table 3) to conserve the original levels of usually rapidly degrading ATP. For optimal conditions, whole blood samples were collected in EDTA tubes and immediately treated with the inhibitory solution. Then plasma was separated and stored at -80°C until further analysis.

**Liquid chromatography–tandem mass spectrometry (LC-MS/MS)-based targeted metabolomics**

Plasma samples were thawed on ice and spiked with 13C6-nicotinamide (Sigma-Aldrich), as an internal standard. Metabolites were extracted by mixing the sample with pre-cooled acetonitrile/water 8:2 (v/v) and vortexing thoroughly at the highest level for 1 minute. Following homogenization, samples were incubated for 10 minutes on ice and centrifuged at 4°C and 16,000 x g for 10 minutes. The supernatants were transferred to a brown glass vial (Phenomenex) and tightly closed with a crimped cap (LABSOLUTE®). The extracts were measured immediately after extraction using a triple quadrupole mass spectrometer (QTRAP® 5500; SCIEX) coupled to an ultra-high-pressure liquid chromatography system (Nexera X2; Shimadzu). Analytes were separated by HILIC using a Luna® HPLC column (3 µm NH2 100 Å 150 mm x 2 mm, Phenomenex). Solvent A contained 20 mM ammonium acetate in MS-grade water (pH 9.8) and solvent B contained 100% acetonitrile. The flow rate was set to 0.25 mL/min with a linear gradient of decreasing solvent B (0-17 min, 80-0%), followed by 8 minutes at 0% solvent B and 5 minutes re-equilibration at 80% solvent B. The MS was equipped with an ESI source and all measurements were carried out in negative ionization and multiple reaction monitoring (MRM) scan mode. Data analysis was performed using SCIEX OS software and by manual quantification in Microsoft Excel. Analyte peaks were identified based on MRM transitions and retention times. A standard calibration curve ranging from 2 nM to 20 µM was used for accurate quantification and all analyte peak areas were normalized against internal standard peak areas.

**Detection of extracellular DNA**

Extracellular DNA was detected in plasma samples diluted 1:5 in HBSS-/0.2% BSA-/ 4 mM EDTA. Sytox Green (S7020, Life Technologies) was added to reach a final concentration of 1 μM/ well. Fluorescence was recorded after 5 min incubation (Tecan Spark 10M).

**Neutrophil isolation**

Blood was layered on Histopaque 1119 (Sigma-Aldrich) and centrifuged at 800 × g for 20 minutes to collect the neutrophil-rich layer. Cells were rinsed with Hanks-buffered salt solution without divalent cations (HBSS; #14170112, Life Technologies) supplemented with 5 mM EDTA and 0.1% bovine serum albumin (BSA; #A1470, Sigma-Aldrich). For further fractionation, a discontinuous Percoll gradient (1.130 g/ml density, #17089101, Cytiva) was employed. After centrifugation at 800 × g for 20 minutes, the resultant neutrophil-rich layer was collected.

**Immunofluorescence analysis of NET formation *in vitro***

Stimulated neutrophils were seeded on poly-l-lysin (#P4707, Sigma-Aldrich) coated coverslips for 4 hours at 37°C and fixed with 4% PFA at 4°C overnight. Permeabilization was performed by using 0.5% TritonX-100 (#T9284, Sigma-Aldrich) for 30 minutes. Primary antibodies including chromatin (anti-histone H2A/H2B/DNA-complex, 5mg/ml, Davids Biotechnologie GmbH), myeloperoxidase (MPO; 1:100, Abcam Cat# ab9535), neutrophil elastase (NE; 1:100, Abcam Cat# ab21595) and CitH3 (10ug/ml, Abcam Cat# ab5103) were applied for 1 hour at RT. Secondary antibodies (Alexa Fluor 647 goat anti-rabbit (1:500, Abcam Cat# ab150079) and Alexa Fluor 594 donkey anti-mouse (1:200, #JIM-715-585-150, Biozol)) were incubated for 1 hour at RT, followed by sealing with DAPI mounting medium (#F6057, Sigma-Aldrich).

**Immunofluorescence NET staining of human stroke brain tissue**

Paraffin sections were deparaffinized and rehydrated, followed by boiling in antigen retrieval buffer (sodium citrate pH 6.0) for 20 minutes. Sections were then blocked in 2% BSA and 0.1% Triton X-100 for 45 minutes at RT. The primary antibody was applied overnight at 4°C. After washing three times with PBS, sections were incubated with secondary antibodies (Alexa Fluor 594 donkey anti-rabbit (1:200, Thermo Fisher Scientific, Cat# A32754) and Alexa Fluor 488 donkey anti-mouse (1:200, Thermo Fisher Scientific, Cat# A32766)) for 60 minutes at RT. After final washing, sections were covered with Fluoromount containing DAPI (#F6057, Sigma-Aldrich).

**Supplementary Tables**

**Supplementary Table S1. Demographics and characteristics of the non-stroke control group**

| **Patients’ characteristics** | **Control group (n = 10)** |
| --- | --- |
| Age (years), mean (range) | 53 (21-75) |
| Sex |  |
| Female | 7 |
| Men | 3 |
| Indication for diagnostic angiography |  |
| Aneurysm control | 6 |
| Arteriovenous malformation | 3 |
| other | 1 |

**Supplementary Table S2. Flow cytometry antibodies**

| **Target** | **Clone** | **Conjugate** | **Catalog number** |
| --- | --- | --- | --- |
| Intracellular Cytokine Monocytes Panel | | | |
| CD14 | M5E2 | AF700 | BioLegend Cat# 301822 |
| CD20 | 2H7 | APC/Fire^TM^ | BioLegend Cat# 302357 |
| CD123 | 6H6 | PE-Cy7 | BioLegend Cat# 306010 |
| HLA-DR | L243 | BV785^TM^ | BioLegend Cat# 307642 |
| IL-1β | H1b-98 | Pacific Blue^TM^ | BioLegend Cat# 511710 |
| IL-8 | E8N1 | AF488 | BioLegend Cat# 511412 |
| IL-10 | JES3-9D7 | PE | BioLegend Cat# 501404 |
| IL-12/ IL-23 p40 | C11.5 | PerCP-Cy5.5 | BioLegend Cat# 501821 |
| TNFα | Mab11 | BV650 | BD Biosciences Cat# 563418 |
| Live/Dead | N/A | Pacific Orange^TM^ | ThermoFisher Scientific #P30253 |
| ASC Speck Monocytes Panel | | | |
| CD14 | M5E2 | APC | BD Biosciences Cat# 561383 |
| CD33 | WM53 | BV421 | BioLegend Cat# 303416 |
| ASC | HASC-71 | PE | BioLegend Cat# 653904 |
| Neutrophil Panel | | | |
| CD14 | MφP-9 | V450 | BD Biosciences Cat# 560349 |
| CD16 | 3G8 | BV605 | BioLegend Cat# 302040 |
| CD45 | 2D1 | BV785 | BioLegend Cat# 368528 |
| Live/Dead | N/A | Pacific Orange^TM^ | ThermoFisher Scientific #P30253 |

**Supplementary Table S3. ATP inhibitory mixture**

| **Reagent** | **Catalog number** | **Final concentration** |
| --- | --- | --- |
| EDTA solution (pH 8.0) | Applichem #A3145.0500 | 15.000 µM/L |
| 1x D-PBS (w/o MgCl₂, CaCl₂) | Gibco #14190-094 | N/A |
| EHNA hydrochloride | Cayman Chemicals #13352 | 100 µM/L |
| 5-Iodotubericidin | Merck #I100 | 100 µM/L |
| Dipyridamole | Merck #D9766 | 40 µM/L |
| ARL 67156 trisodium salt | Tocris Bioscience #1283 | 150 µM/L |
| Adenosine 5'-(α,β-methylene) diphosphate sodium salt | Tocris Bioscience #3633 | 220 µM/L |
| S-(4-Nitrobenzyl)-6-Thioinosine | Merck #N2255 | 100 µM/L |

**Supplementary Figures**

**Supplementary Fig. 1: Single-cell quality control plots in arterial and ischemic blood samples.** (A) Violin plots displaying nFeature RNA, nCounts RNA, the percentage of mitochondrial genes (percent.mt), the percentage of ribosomal genes (percent.ribo) and the percentage of heat shock protein genes (percent.hs). (B) Bar plots summarizing the distribution of major cell populations across conditions. (C) UMAP projection based on RNA-derived integration embeddings.

**Supplementary Fig. 2: Immune cell profiling of arterial and ischemic blood.** (A) UMAP plot showing clusters and annotation of B cells identified in ischemic and non-ischemic arterial blood. (B) UMAP plot showing clusters and annotation of T cells identified in ischemic and non-ischemic arterial blood. (C) UMAP plot showing clusters annotation of NK cells identified in ischemic and non-ischemic arterial blood. (D) Dot plot showing the RNA expression levels and frequency of cell surface protein markers within NCLM, IMM, and CLM from arterial versus ischemic blood samples. (E) Dot plot and (F) violin plots showing the expression levels of CD88 on annotated immune cell populations and on NCLM, IMM, and CLM from arterial versus ischemic blood samples (including median and interquartile range). (G) Density UMAP analyses showing the protein expression levels of cytokines by NCLM, IMM, and CLM from arterial versus ischemic blood samples. P-values were determined using a Wilcoxon matched-pairs signed-rank test (***P* < 0.01).

**Supplementary Fig. 3: Inflammatory immune mediator plasma levels stratified by sex in ischemic blood samples.** Plasma concentrations of inflammatory mediators (A) IL-1β (*n* = 27), (B) IL-8 (*n* = 29), (C) CXCL1 (*n* = 29), (D) CitH3 (*n* = 33) and (E) extracellular DNA (exDNA; *n* = 40) are shown for men and women. Data are shown as median (IQR); n.s. = not significant. Statistical analysis was performed using the Mann-Whitney U test for unpaired samples.

**Supplementary Fig. 4: Inflammatory immune mediator plasma levels in patients with and without**

**rtPA treatment.** Plasma concentrations of inflammatory mediators (A) IL-1β (*n* = 27) and (B) CitH3 (*n* = 33) were quantified in acute ischemic stroke patients stratified by intravenous rtPA treatment. Data are shown as median (IQR). **P < 0.01, ***P < 0.001, and ****P < 0.0001. Statistical analysis was performed using the non-parametric Wilcoxon signed-rank test for paired comparisons.

**Supplementary Fig. 5: Intracellular cytokine expression in monocytes.** Exemplary FACS plots displaying varying cytokine expression of IL-1β, IL-8, TNFα, IL-10 and IL-12 after restimulation with 100 ng/ml IFNγ and 1.25 µg/ml LPS in (A) ischemic, (B) matched non-ischemic arterial and (C) venous blood samples from one stroke patient.

**Supplementary Fig. 6: NET markers are already elevated within the first 4.5 hours after stroke onset.** CitH3 (*n* = 12) levels in the ischemic blood plasma compared to matched non-ischemic arterial plasma with an overall maximum ischemia time of 4.5 hours. Statistical analysis was performed using the non-parametric Wilcoxon signed-rank test for paired comparisons. Data shown as median (IQR). ****P* < 0.001.

**Supplementary Fig. 7: Association between stroke severity and serum biomarkers.** Correlation between ASPECTS and ischemia duration and the serum concentrations of (A, B) IL-1β (n = 27), (C, D) IL-1RA (n = 29), and (E, F) IL-8 (n = 29). A linear regression model was used for statistical analysis. The light blue shaded area represents the 95% confidence interval. Ischemia time was missing for (B) n = 7, (D) n = 8, and (F) n = 8.
